# Identifying critical outbreak time window of controversial events based on sentiment analysis

**DOI:** 10.1371/journal.pone.0241355

**Published:** 2020-10-29

**Authors:** Mingyang Wang, Huan Wu, Tianyu Zhang, Shengqing Zhu

**Affiliations:** 1 College of Information and Computer Engineering, Northeast Forestry University, Harbin, People’s Republic of China; 2 Institute of Image Processing and Pattern Recognition, Shanghai Jiaotong University, Shanghai, People’s Republic of China; Universitat de Barcelona, SPAIN

## Abstract

The response of netizens toward controversial events plays an important guiding role in the development of events. Based on the identification of such responses, this study aimed to determine the critical outbreak time window of events. The microblog texts related to an event were divided into seven emotional categories via multi-emotional analysis to capture the subtle emotions of netizens toward an event, i.e., public opinion. By detecting the characteristics of the text and regional coverage of emotions, an emotional coverage index that reflects the intensity of emotional impact was proposed to determine the mainstream emotion of netizens. By capturing the mutation characteristics of the impact intensity of mainstream emotions, the critical time window of the public opinion toward the event was obtained. The experimental results demonstrated that the proposed method can effectively identify the critical outbreak time window of controversial events, which can help authorities in preventing the further aggravation of events.

## Introduction

The upsurge of public opinion in controversial events severely threatens the social order and endangers public security. If the critical time window before the outbreak of public opinion can be determined in a timely manner, it will help authorities in identifying the problems reflected by the public opinion to take appropriate measures that can reduce the adverse social impact due to the further development of public opinion. With the popularity of mobile internet, new media based on the internet and social network platforms has gradually replaced traditional media and has become a mainstream form of media [1]. As a type of new media, microblogs have been developed and popularized rapidly in China [2]. An increasing amount of news and opinions are posted on Sina Weibo by the media and public, thereby making it an important platform for Chinese netizens to release public opinions [3]. In a series of controversial events in recent years, the network public opinion due to new media platforms such as microblogs has played an important role [4, 5]. Thus, the analysis of public opinion data related to controversial events on new media platforms can help in better capturing the development trend of events and provide valuable auxiliary support to the authorities to cope with the impact of the development of an event.

Researchers have stated that the response of netizens toward the public opinion of an event is an important factor affecting the evolution of the event. They pointed out that the emotional strength of netizens changes during the evolution of an event [6], and argued that the formation of online public opinion is impacted by the change in netizens’ views [7–9]. They also pointed out that emotions aroused by the online participation of the public on social media will further affect their online behavior [10]. Consequently, based on the cognitive appraisal theory [11], a cognition-emotion behavior process was proposed to explain this phenomenon [12]. The process shows that the appraisal of events can trigger certain emotional reactions [13] that can affect the behavioral intentions of an individual [14]. To detect the emotions of netizens toward events on social media, researchers constructed various emotional dictionaries [15–18] and established different machine learning models [19–24].

In conclusion, emotion plays an important role in guiding the development of the public opinion toward events. Moreover, different emotional categories have different impacts on the development of public opinion. Some researchers have pointed out that in the process of public opinion dissemination, the impact of negative emotions is often greater than that of positive emotions. This conclusion can be drawn even through the case study of positive events [25]. However, there are few studies that examined the role of negative emotions in public opinion early warning. In other words, few studies have predicted the outbreak of public opinion by monitoring the evolution of negative emotions. This paper presents a quantitative and in-depth study on this issue. By identifying the mainstream negative emotions that affect the development of public opinion, combined with the characteristics of the text and regional coverage of mainstream negative emotions, this study discusses the value of emotions in early warning of public opinion toward an event.

The contribution of this study is threefold. First, based on the examination of specific emotions expressed by emoticons, the microblog texts related to an event are divided into multiple emotions to obtain the subtle emotions of netizens for the event. Second, based on the investigation of the characteristics of text coverage and geographical coverage of different emotions, the mainstream negative emotions that play a key role in guiding the development of the public opinion toward an event are determined. Third, the critical time window before the outbreak of public opinion is determined by tracking the mutation of the intensity characteristics of mainstream negative emotions. The mutation of the intensity of mainstream negative emotions is an important signal for public opinion warnings. This mutation shows that the social contradictions reflected by the mainstream negative emotions have reached the edge of intensification and outbreak. Therefore, the time window of the sudden change in the intensity of mainstream negative emotions is the key time window before the outbreak of public opinion. After this time window, if the authorities do not implement timely and reasonable policies to intervene and control the public opinion toward an event, the event will quickly evolve into a more controversial event, which will cause a more malignant impact on the society and lives of the people to a greater extent.

In the next section, we present a literature review of the related works before describing the detailed method for identifying the critical outbreak time window of controversial events. We then present the experimental results and discussion to verify the proposed method. Finally, we summarize our work and provide future research directions.

### Literature review

When an event occurs, social media platforms are often used as a channel by people in different parts of the world to express their views, feelings, and comments [26]. Consequently, the social media content released during the evolution of an event is increasingly used by news media and researchers to describe the public response to these events [26]. In this section, we summarize related research.

Researchers believe that social media plays an important role during the upsurge of controversial events [27]. Through its inherent multi-channel communication channels and highly interactive characteristics, social media has a tremendous impact on the event as well as the understanding of netizens toward the event [28, 29]. During the evolution of an event, social media may potentially influence the thoughts, behaviors, and reactions of people [30–33]. According to a telephone survey in China, 64.7% of the respondents participated in discussions and communication related to controversial events, and more than 40% participated in social media communication [34]. Thus, it can be concluded that social media users are no longer passive recipients of information, but active participants in event communication [35].

Emotions play a special role in the evolution of events, leading to certain intentional behaviors in social media. A fine-grained emotional analysis was proposed for Twitter to better capture the emotions of netizens during event management [36]. Some researchers proposed that the emotion strength of Twitter users may change during an emergency. They found that in the process of public opinion dissemination, negative emotions usually play a greater role than positive emotions, even for positive events [25]. Researchers also noted that people’s emotions toward an event change over time [37]. To detect the response of netizens toward events, researchers have constructed different emotional dictionaries for different social media platforms [15–18]. Various techniques of machine learning [19–24], deep learning [38–40], and natural language processing [41, 42] are also widely used in the emotional analysis of event-related texts [43–46].

In addition, researchers have discussed the regional emotional bias in the evolution of public opinion toward an event [47]. They explored the emotional prejudices in different language regions on the same event and found some interesting observations. For example, in the 2016 terrorist attacks in Paris, 16% more negative comments were written in English than in French, even though the incident originated in France [48]. Researchers also studied the communication between citizens and local governments during 18 snowstorms in Maryland, USA, to potentially help local governments determine the informational requirements of citizens [49].

These studies show that different emotions of netizens on social media will resonate differently among the public, and thus have different impacts on the development of events. However, we still lack a quantitative perspective to test whether there is a dominant emotion that can guide the development of public opinion and if the evolution of such a dominant emotion can help in issuing early warnings before the outbreak of public opinion.

This study aims to identify the specific dominant emotions that play a major role in the development of events and explore the early warning effect of these emotions on the outbreak of public opinion. In contrast to previous studies, this study quantitatively analyzes the influence of emotion. By quantifying the text coverage and regional coverage characteristics of different emotions, we identify the mainstream emotions that play a leading role in the development of public opinion toward an event. By detecting the mutation of the impact intensity of mainstream emotions, we can find the time window of the emotional outbursts of netizens. The time window is the key time point before the outbreak of public opinion, which provides direct guidance for issuing early warnings related to public opinion.

## Methods

**Fig 1:**
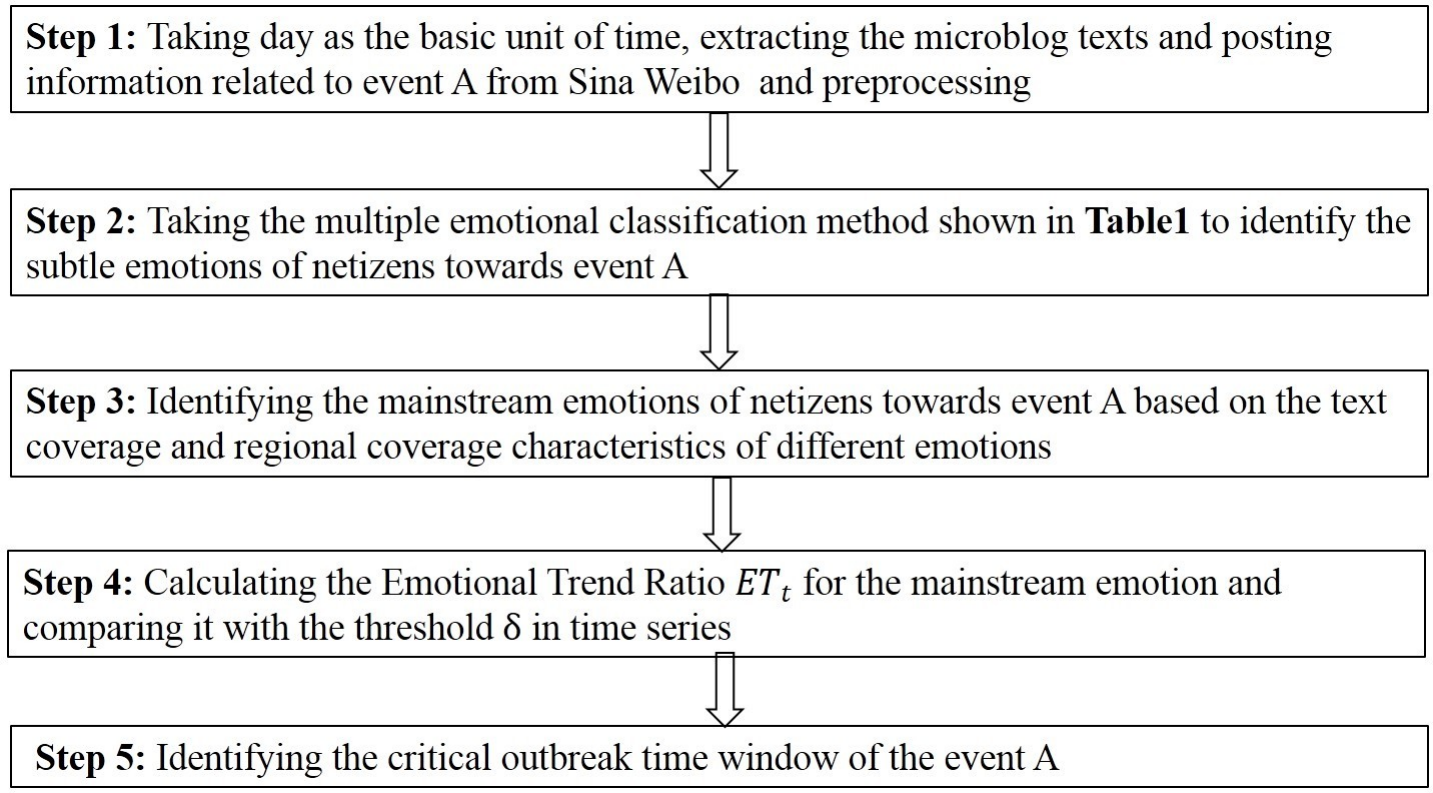
Schematic diagram of the process of identifying the critical outbreak time window.

This study mainly identifies the critical outbreak time window of the public opinion toward an event by monitoring the evolution of the emotions of netizens. Among them, identifying the mainstream negative emotions of netizens and monitoring the change in the impact intensity of these emotions are the key issues. This section provides a detailed process to solve the aforementioned problems. Taking event ***A*** as an example, Fig 1 shows a schematic diagram of the method proposed in this paper.

There may be some special symbols in the original microblog texts that are not valuable for our experiments. Thus, before performing the following experiments, we first removed all unnecessary symbols from the collected microblog texts.

1. The web link URL and HTML format code that appear in the microblog texts were filtered out.
2. The forwarding tag and username after the symbol “@” were deleted to avoid the possible influence of these tags on the following emotional analysis.
3. The symbols “#” and the data between them were deleted because they are basically unrelated to the event.
4. The Jieba package in Python was used to segment the microblog texts after completing the above filtering operations. Furthermore, there are still some words, such as modal words and function words, which are not helpful in analyzing the texts. They were removed from the word lists to improve the efficiency and quality of text expression. Table 1 shows examples of modal words and function words that were deleted from the microblog texts in this study.

**Table 1:** Modal words and function words deleted from the microblog texts.

Category	Deleted words
modal words	must; may; might; need; dare; can; could; used to; ought to; shall; should; will; would
function words	a; an; the; in; on; from; above; behind; before; at; by; for; of; to; with; onto; into; within; since before; until after; in front of; because of; and; both; also; while; or; that; whether; if; when; as; since; once; as soon as; until; till; because; although; though; even if; even though; no matter how; no matter what; whatever; however; whether… or; hi; oh; ah; alas; if; unless; so long as; as long as; do; did; where; what; who; which; how; why; whose; wherever; whichever; whoever; whenever

### Multiple emotional classification of event-related microblog texts

Netizens express and exchange opinions on social platforms around events, which is essentially an emotional release of public opinion [50, 51]. Emotional analysis can identify the attitudes, thoughts, judgments, and emotions of netizens toward an event through event-related text data [24, 52].

Researchers have constructed emotional dictionaries for Twitter, Weibo, and other platforms by considering the official language and linguistic expression habits of different social media platforms [15–18, 53]. The emotional dictionary used for a microblog platform mainly relies on open source emotional dictionaries, including HowNet [54], the Chinese emotional vocabulary ontology database of the Dalian University of Technology [55] and NTUSD of the National Taiwan University [56]. In addition to emotional words, netizens often express their emotions via other symbols, such as emoticons, slangs, and hashtags. Researchers have widely discussed the role of these features in identifying the emotions of texts on Twitter [57–63]. Among them, emoticons, which have clear emotional meanings, are often widely used by netizens in microblog environments to express their feelings [64–66]. They are important emotional signals for microblog sentimental analysis.

In previous study, by summarizing the emoticons used by microblog users to express their specific emotions, we extracted emotional words from the sentences covered by each emoticon [67]. The emotional words extracted based on a given emoticon were considered to have the same emotional tendency as the emoticon. They were finally used to expand the Chinese emotional vocabulary ontology database proposed by the Dalian University of Technology. The vocabulary includes seven emotional categories of “Happiness,” “Like,” “Sadness,” “Disgust,” “Astonishment,” “Anger,” and “Fear.” In comparison to the traditional two or three types of emotions, the seven emotional categories can better express the subtle feelings of netizens toward an event. In this study, the expanded emotional dictionary was introduced to determine the emotions of netizens toward events. Simultaneously, the deep learning model, Word2vec, was used to vectorize the emotional words, followed by the vectorization of the sentences where the emotional words were located. Because Word2vec can capture the contextual information of emotional words in a microblog corpus [68], the emotional word vector and sentence vector trained by Word2vec can better express the semantic information of texts, thereby improving the performance of identifying the emotions of netizens. Table 2 presents the algorithm for classifying the event-related microblog texts into seven-element emotions.

**Table 2:** Seven-element emotion classification algorithm on event-related microblog texts.

Algorithm: Seven-element emotion classification algorithm on the event-related microblog texts
Input: Event-related microblog text corpus; Expanded emotional dictionary; Dictionary of negative words; KNN classifier
Output: Event-related microblog text corpus with seven-element emotions
Steps:
1) Data preprocessing;
2) Searching for emotional words in each microblog text by using expanded emotional dictionary;
3) Training the emotional words into 50-dimensional vectors by Word2vec model based on the event-related microblog text corpus;
4) Determining the negative words vector of the microblog text by examining the microblog text with the negative words dictionary. If a negative word appears in the text, the negative vector is 1; otherwise, it is 0.
5) The vectors of emotional words appearing in the same micro-blog text were added linearly, and the result was combined with the negative words vector of the micro-blog text to form the final 51-dimentional text vector.
6) Using KNN Classifier to classify the texts into seven-element emotions.

### Identifying critical outbreak time window

In different stages of the public opinion dissemination of an event, netizens express their feelings by posting comments about the event. Different emotions have different guiding effects on the evolution of events. Generally, negative emotions are more likely to resonate with netizens, and it plays a more important role in guiding the development of events [51]. Therefore, in the study of evolutionary characteristics of the public opinion toward an event, researchers focused on the identification of negative emotions [69–71]. However, this type of exploration is more concerned with the number of texts covered by negative emotions, while ignoring the regional characteristics of negative texts. In addition to the text, the geographic characteristics of netizens are also a very important feature that can reflect the scope of negative emotions in a region [46]. If a negative emotion extensively and evenly covers a large amount of area, it will further lead to more resonance among the netizens, thereby further stimulating the development of the negative influence of events. Some researchers recognized the importance of regional characteristics and incorporated the region dimension into the discussion of the development trend of events [72–74]. However, most of these studies focus on the discussion of local events based on the regional characteristics, without the discussion of mainstream negative emotions and public opinion outbreak warning.

In this study, both the text and geographical coverage characteristics of different emotions were considered to identify the mainstream negative emotions affecting the evolution of events. Considering city as the basic geographical unit, the geographic data of netizens when posting comments to an event was collected. Because the netizen response to such events is dynamic during different periods of an event, the event-related microblog texts were divided into different time windows based on the day as the basic unit of time. By analyzing the text and geographical coverage characteristics of each emotion in different time windows, the negative emotions with higher coverage in maximum time windows were identified as the mainstream negative emotions. The changes in the coverage characteristics of the mainstream negative emotions reflect the changes in attitudes and opinions of netizens in different regions. If the coverage of mainstream negative emotions suddenly surges, it implies that the social contradictions reflected by these emotions have aroused more resonance among netizens, which, to a large extent, will lead to a concentrated outbreak of social contradictions reflected by events. Therefore, the evolutionary characteristics of mainstream negative emotions play a more important role in guiding and predicting the development trend of events. Based on the identification of mainstream negative emotions, this study focuses on the mutation point of these emotions on the time axis, and considers the time window of the mutation of mainstream negative emotions as the critical time window before the event outbreaks.

Several definitions are given before providing the algorithm for identifying the critical outbreak time window. In the following definitions, symbol *t* is used to represent the time window with a value ranging from *1* to *n*. *n* denotes the total number of time windows divided by the current event; that is, the duration of the event from germination to extinction. Symbol *i* denotes the emotion with a range of values ranging from *1* to *7*.

#### Definition 1

Text coverage ratio *TCR*_*ti*_: Calculates the ratio of the number of event-related microblog texts, *TC*_*ti*_, covered by emotion *i* to the total number of texts, *TC*_*t*_, in each time window.

$$TCR_{ti}=\frac{TC_{ti}}{\left(TC_{t}+\beta \right)}$$ (1)

The total number of texts, *TC*_*t*_, in one time window is the sum of texts covered by each emotion in that time window; that is ${TC}_{t}=\sum_{i=1}^{7}{TC}_{ti}$.

In Formula (1), a parameter *β* is added to the denominator to eliminate the data sparsity that may exist in a given time window. For example, if the netizens only post one microblog text in one time window, the text coverage ratio of the emotion category that the text is classified into would reach the maximum; that is, *TCR*_*ti*_ = 1. However, this situation has no statistical significance, and the value of *TCR*_*ti*_ calculated in this time window is not valuable for exploring the evolutionary characteristics of events. By observing the daily posts of the four events used in this experiment, we found that the maximum number of posts per day was just over 5000. Therefore, we added a sparsity parameter, *β* = 5000, to the denominator to eliminate the possible impact of data sparsity on the emotional analysis.

#### Definition 2

Regional coverage ratio *RCR*_*ti*_: it calculates the ratio of the number of regions, *RC*_*ti*_, covered by each emotional category to the total number of regions, *RC*_*ti*_, covered by all emotions in each time window.

$$RCR_{ti}=\frac{RC_{ti}}{RC_{t}}$$ (2)

The total number of regions, *RC*_*ti*_, covered by different emotions in one time window is the sum of the number of regions covered by each emotion; that is, ${RC}_{t}=\sum_{i=1}^{7}{RC}_{ti}$.

The text coverage ratio, *TCR*_*ti*_, and regional coverage ratio, *RCR*_*ti*_, give the proportion of texts and regions covered by different emotions in different time windows. Evidently, higher values of *TCR*_*ti*_ and *RCR*_*ti*_ mean that a particular emotion was dominant among the netizens, thereby demonstrating that netizens from different regions express the same emotions about events by posting comments on social media platforms. However, the regional coverage ratio, *RCR*_*ti*_, can only measure the geographical scope of emotional radiation and cannot express the uniformity of emotions in the geographic coverage. If a certain emotion has higher coverage and more uniform distribution in these areas, it can better explain that this emotion has been more widely recognized among netizens in different areas, and this emotion can be considered as the mainstream emotion of netizens in different areas.

To calculate the regional distribution uniformity of different emotions, we calculated the regional distribution index of emotions. The regional distribution index refers to the ratio of the number of texts covered by one emotion posted in each region to the total number of texts covered by the same emotion in each time window. Accordingly, the information entropy is calculated to measure the regional diffusion uniformity of different emotions.

#### Definition 3

Regional distribution *RD*_*tij*_: In each time window, the number of event-related texts, *TC*_*tij*_, covered by emotion *i* in each different area accounts for the proportion of the total number of event-related texts, *TC*_*tij*_, covered by emotion *i* in the same time window.

$$AD_{tij}=\frac{TC_{tij}}{TC_{ti}}$$ (3)

*TC*_*tij*_ represents the number of microblog texts covered by emotion *i*, posted in area *j* under the *t*-th time window. *TC*_*ti*_ represents the total number of microblog texts covered by emotion *i* that were posted under the *t*-th time window.

Based on the regional distribution, *RD*_*tij*_, of each emotion, the regional diffusion uniformity under different time windows was calculated by the information entropy.

#### Definition 4

Regional coverage information entropy *ERD*_*ti*_: Calculate the uniformity of the geographic coverage of each emotion in each time window.

$$ERD_{ti}=-\sum_{j}AD_{tij}logAD_{tij}$$ (4)

The value range of *ERD*_*ti*_ is [0,1]. The larger the index value, the more evenly distributed the netizens are with this emotion; this implies that the emotion resonated among the netizens in different regions. The smaller the index value, the netizens are relatively concentrated in a few regions, indicating that such emotions have high regional concentration characteristics and will not cause large-scale diffusion. Comparatively, the larger the value of the index, the more significant the emotion will be in guiding the development of public opinion.

Combining the text coverage ratio, *TCR*_*ti*_, regional coverage ratio, *RCR*_*ti*_, and regional coverage information entropy, *ERD*_*ti*_, of different emotions, this study proposes a comprehensive coverage index of emotions to measure the comprehensive impact intensity of emotions in different time windows. The values of, *TCR*_*ti*_, *RCR*_*ti*_, and *ERD*_*ti*_ are all between [0,1]. If these indicators are combined in an additive way, it may be difficult to clearly demonstrate the difference in the comprehensive coverage of each emotion. Therefore, in this study, these indicators were multiplied to highlight the differences in the comprehensive coverage of different emotions.

#### Definition 5

Emotional coverage *EC*_*ti*_: In each time window, the text coverage ratio, *TCR*_*ti*_, regional coverage ratio, *RCR*_*ti*_ and regional coverage information entropy, *ERD*_*ti*_ of different emotions are synthesized to obtain the comprehensive coverage value of each emotion.

$$EC_{ti}=TCR_{ti}*RCR_{ti}*ERD_{ti}$$ (5)

In a certain time window, if an emotion covers more event-related texts and has higher geographical coverage and higher geographical distribution uniformity, it implies that the emotion is resonated by more netizens from different regions. This type of emotion can be taken as the mainstream emotion of netizens in this time window and it will play an important guiding role in the evolution of public opinion.

#### Definition 6

Mainstream emotion: The mainstream emotion is the emotion with the largest value of emotional coverage, *EC*_*ti*_.

When the negative mainstream emotion mutates in a certain time window, its direct representation is that when the emotional coverage, *EC*_*ti*_, of this emotion suddenly rises, the subsequent time window will be accompanied by an outbreak of events. The sudden increase in the emotional coverage, *EC*_*ti*,_ of negative mainstream emotion means that this type of emotion has increased sharply at the social level. This surge may induce the same emotional resonance among more netizens, which will lead to the evolution of events into a controversy and have a more adverse impact on the social life. To capture the time window of the surge in the comprehensive emotional coverage of mainstream emotion and obtain the critical outbreak time window before the outbreak of events, the concept of emotional trend ratio is proposed in this paper.

#### Definition 7

Emotional trend ratio *ET*_*t*_: Calculate the ratio of the emotional coverage of mainstream emotion in the current time window to the average of the emotional coverage of the emotion in all previous time windows.

$$ET_{t}=\frac{EC_{t}}{\bar{EC_{i}}}$$ (6)

*EC*_*t*_ indicates the comprehensive emotional coverage of the mainstream emotion in the current time window *t* and $\overset{-}{{EC}_{i}}$ represents the average emotional coverage of the mainstream emotion in all previous time windows.

By observing the value of the emotional trend ratio in the evolving process of events, we found that there exists a threshold that can monitor the outbreak of events. When the emotional trend ratio of the negative mainstream emotion is greater than the threshold in a certain time window, the public opinion toward an event breaks out. Based on this observation, we set the current time window when *ET*_*t*_ begins to exceed the threshold as the critical outbreak time window before the outbreak of the events.

#### Definition 8

Critical outbreak time window *T*_*critical*_: When *ET*_*t*_ begins to exceed the threshold δ, that is *ET*_*t*_ > δ, the current time window is defined as the critical outbreak time window of the events.

It is critical for governments to implement strategies to supervise events before their outbreak to reduce their negative consequences. Table 3 presents the algorithm for identifying the critical outbreak time window of events.

**Table 3:** Algorithm to identify the critical outbreak time window of events.

Algorithm: Identifying the critical outbreak time window of events
Input: Event-related micro-blog texts categorized by seven-element emotions
Output: The critical outbreak time window of events
Steps:
1) Calculating the Text Coverage Ratio TCR_ti_ for each emotion;
2) Calculating the Regional Coverage Ratio RCR_ti_ for each emotion;
3) Calculating the Regional Distribution RD_tij_ and regional coverage information entropy ERD_ti_ for each emotion;
4) Calculating the Emotional Coverage *EC*_*ti*_ based on TCR_ti_, *RCR*_*ti*_ and ERD_ti_.
5) Comparing the Emotional Coverage *EC*_*ti*_ of each emotion in each time window, and taking the emotion with the greatest Emotional Coverage EC_ti_ in most time windows as the mainstream emotion of the event;
6) Calculating the Emotional Trend Ratio *ET*_*t*_ for the mainstream emotion, and comparing it with the threshold δ. When *ET*_*t*_ > δ, the current time window is defined as the critical outbreak time window of the event.

## Experimental results and discussion

### Dataset

Four typical controversial events were selected as examples for our experiments: “Wei Zexi,” “Nanny arson in Hangzhou,” “Mum who steals chicken leg,” and “Luo Yixiao.” Based on these four events, we explored the possibility of identifying the critical outbreak time window before the outbreak of events. All microblog texts related to the four events from the germination to the recession period were collected using a web crawler from the Sina Weibo platform. Data collection was conducted from April 28, 2016 to May 14, 2016; June 22, 2017 to June 30, 2017; May 31, 2016 to June 9, 2016; and November 28, 2016 to December 12, 2016. These four events reflect four different aspects of social life. Among them, the “Wei Zexi” incident aroused concern for “Putian Hospital”; the “Nanny arson in Hangzhou” incident reflects upon the human nature; the “Mum who steals chicken leg” incident reflects the social assistance mechanism; and the “Luo Yixiao” incident makes the netizens reflect on the existing donation mechanism. Considering the length of the revised manuscript, we describe the four incidents in detail in the “S1 File”

## Results and discussion

According to the seven-element emotional classification process given by the algorithm in Table 2, the event-related microblog texts in each time window were divided into seven emotional categories. Considering the “Wei Zexi” incident as an example, we detected the emotional distribution of microblog texts related to the event in different time windows. The results are presented in Table 4. It shows that in different time windows, netizens present different and subtle emotional experiences to events. Under each time window, each emotion had different coverage characteristics on event-related texts.

**Table 4:** Emotional distribution of microblog texts related to the “Wei Zexi” event.

Time window	Anger	Disgust	Fear	Happiness	Like	Sadness	Astonishment
2016/04/28	24	0	0	0	8	37	2
2016/04/29	18	0	0	0	4	18	0
2016/04/30	98	7	1	1	23	99	14
2016/05/01	3143	248	4	46	958	2084	372
2016/05/02	7818	1013	15	83	2462	7162	1410
2016/05/03	11770	993	13	106	3807	9198	1521
2016/05/04	11370	1552	10	91	3277	8080	1334
2016/05/05	8919	706	3	85	2424	5876	1102
2016/05/06	5156	598	5	28	843	4725	823
2016/05/07	2821	324	2	35	598	3131	553
2016/05/08	1935	193	2	29	452	2484	528
2016/05/09	5217	176	1	21	1581	1939	345
2016/05/10	3870	353	4	19	729	2567	422
2016/05/11	2353	127	0	6	227	1757	150
2016/05/12	1294	53	0	4	136	1067	104
2016/05/13	1149	61	0	6	73	815	74
2016/05/14	918	44	1	1	48	548	44

**Fig 2:**
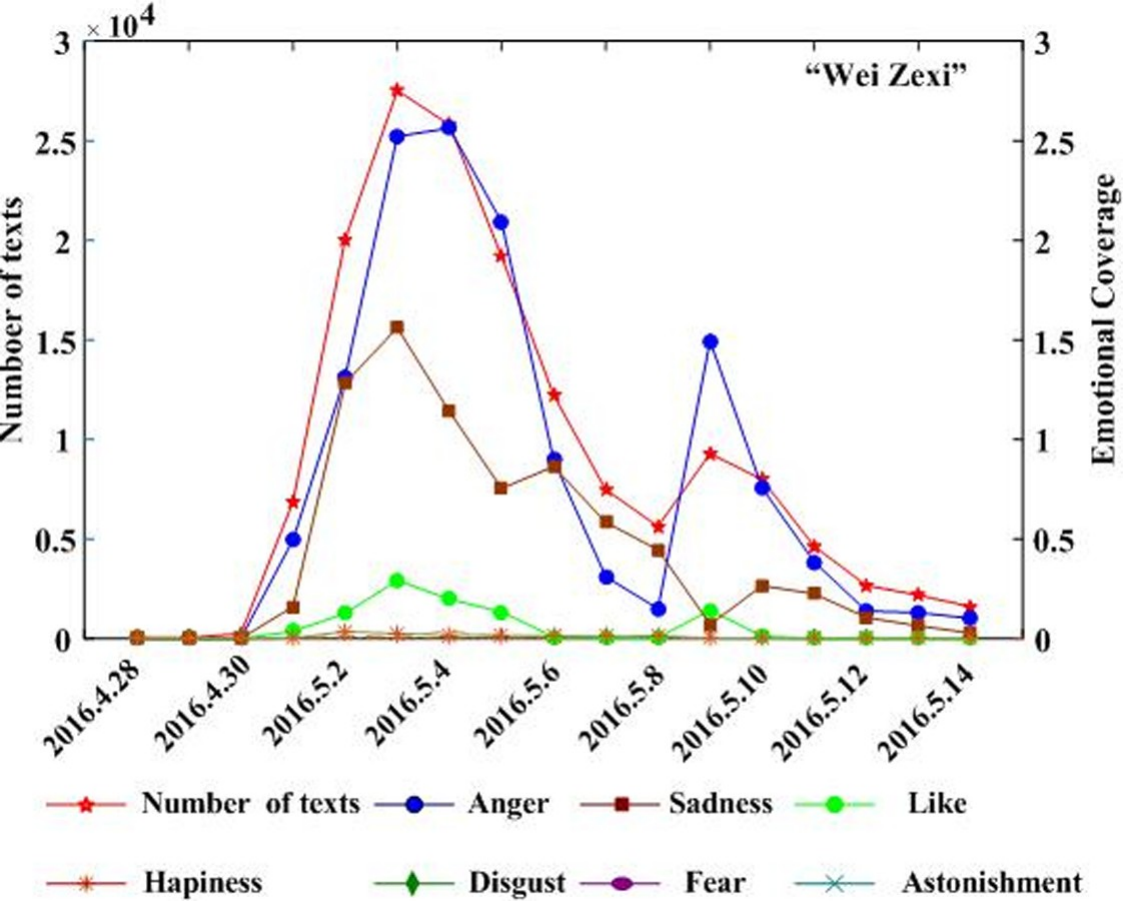
Evolution of emotional coverage of each emotion category for the “Wei Zexi” event.

**Fig 3:**
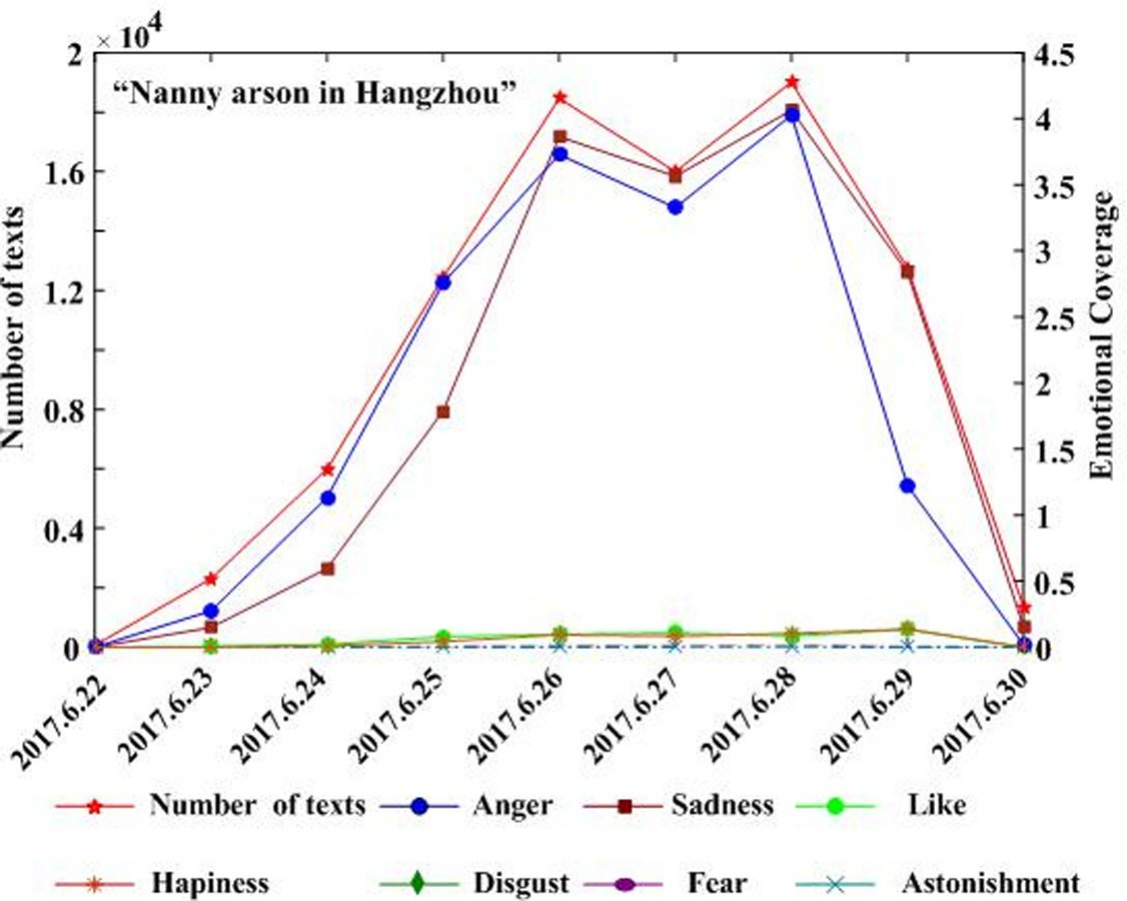
Evolution of emotional coverage of each emotion category for the “Nanny arson in Hangzhou” event.

**Fig 4:**
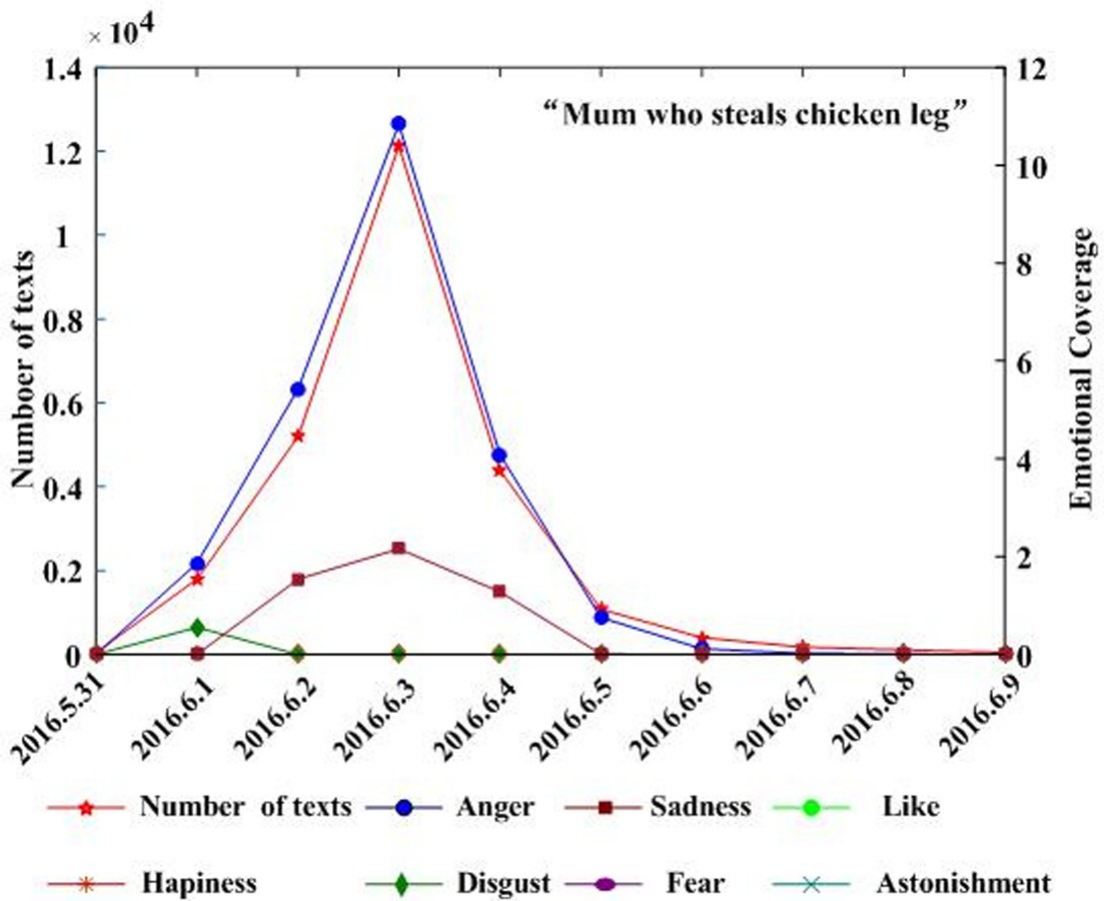
Evolution of emotional coverage of each emotion category for the “Mum who steals chicken leg” event.

**Fig 5:**
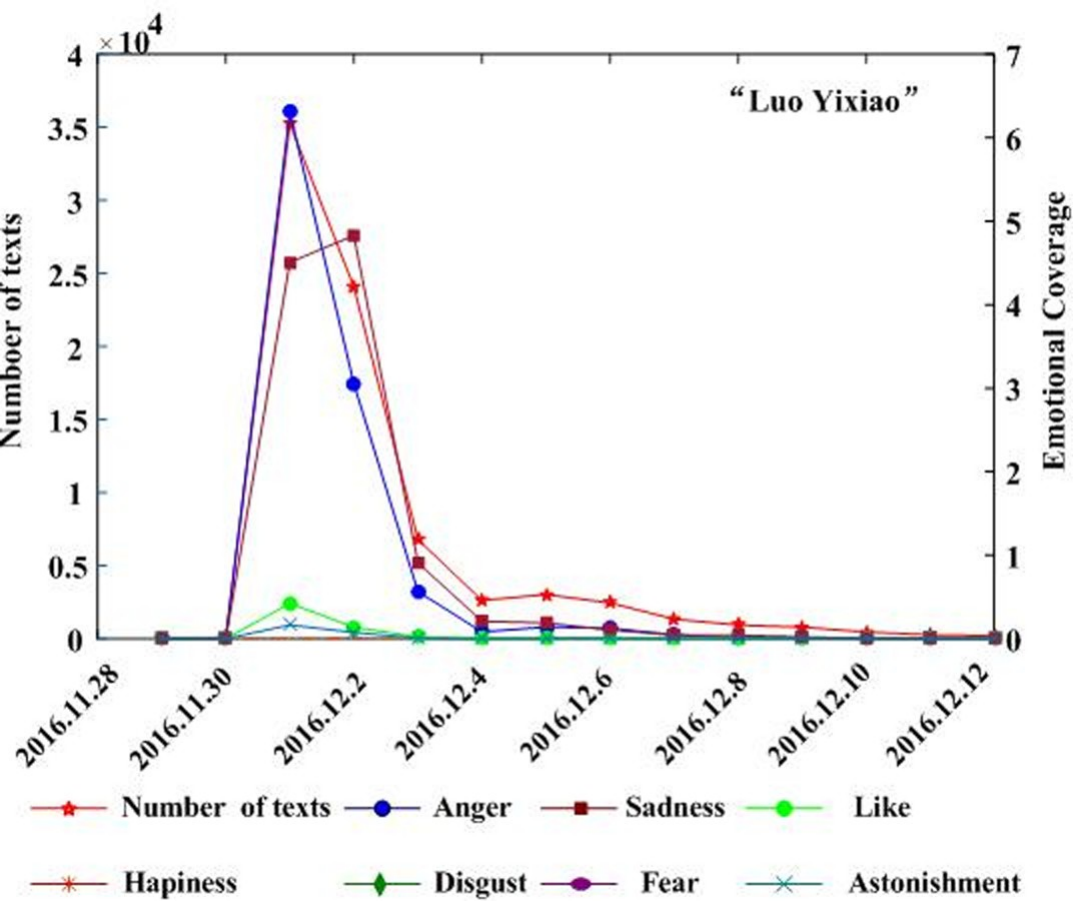
Evolution of emotional coverage of each emotion category for the “Luo Yixiao” event.

Combining the text and regional coverage characteristics of each emotion, we examined the mainstream emotions for the four events. According to the algorithm proposed in Table 3, we calculated the text coverage ratio, *TCR*_*ti*_, regional coverage ratio, *RCR*_*ti*_, regional distribution, *RD*_*tij*_, and regional coverage information entropy, *ERD*_*ti*_, for each emotion. Based on *TCR*_*ti*_, *RCR*_*ti*_, and *RD*_*tij*_, we calculated the emotional coverage, *EC*_*ti*_, for each emotion. Figs 2, 3, 4, and 5 show the evolutionary trend of emotional coverage, *EC*_*ti*_, for each emotion over time for each event. We adopted two y-axes to clearly demonstrate the changes in the coverage of each type of emotion in the process of event evolution. The left y-axis represents the number of event-related microblog texts and the right y-axis represents the value of emotional coverage, *EC*_*ti*,_ under each emotional category. We found that for the four events, the two negative emotions of “anger” and “sadness” always obtained higher values in *EC*_*ti*_ under each time window. These two emotions reflect the attitudes and opinions of most netizens towards an event, and they eventually lead to the outbreak of the event after being dominant among more netizens in a wider range. Therefore, these two negative emotions are the mainstream emotions dominating the development trend of public opinion in these four events. The results are consistent with the conclusions of previous research [25], which show that negative emotions outweigh positive emotions in the process of the public opinion dissemination of an event.

**Fig 6:**
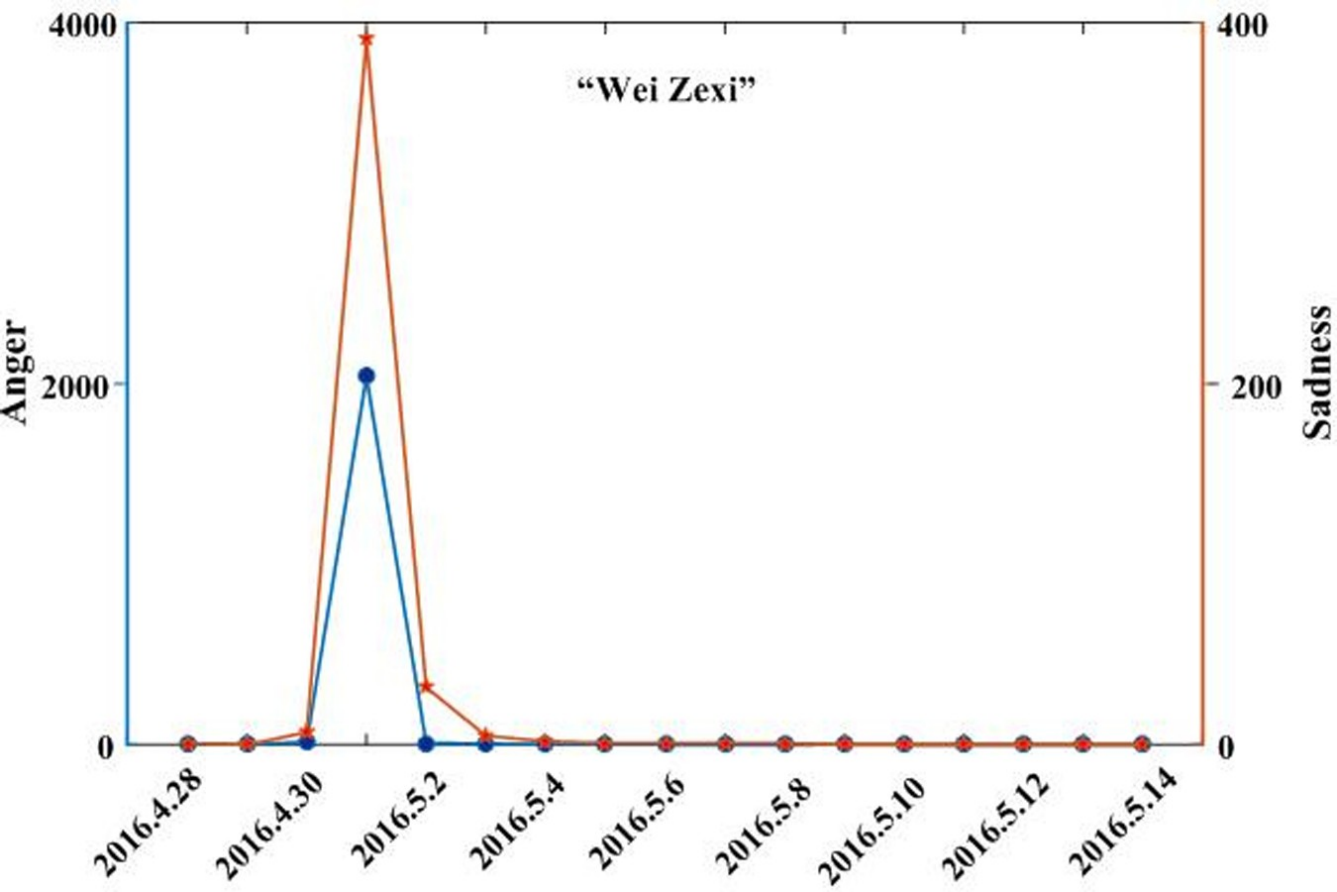
Evolution of emotional trend ratio of the mainstream negative emotions to the “Wei Zexi” event.

**Fig 7:**
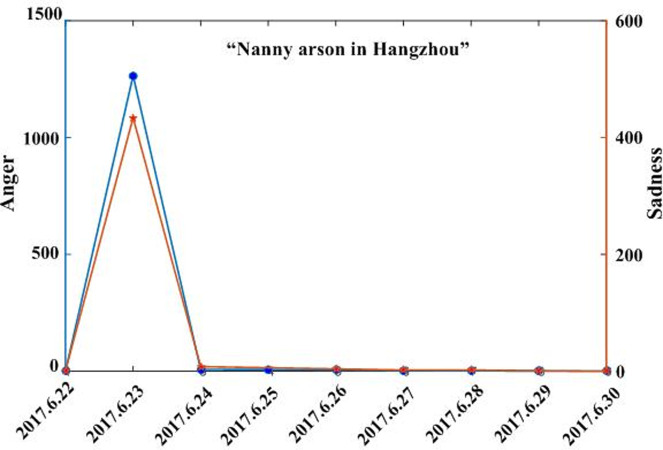
Evolution of emotional trend ratio of the mainstream negative emotions to the “Nanny arson in Hangzhou” event.

**Fig 8:**
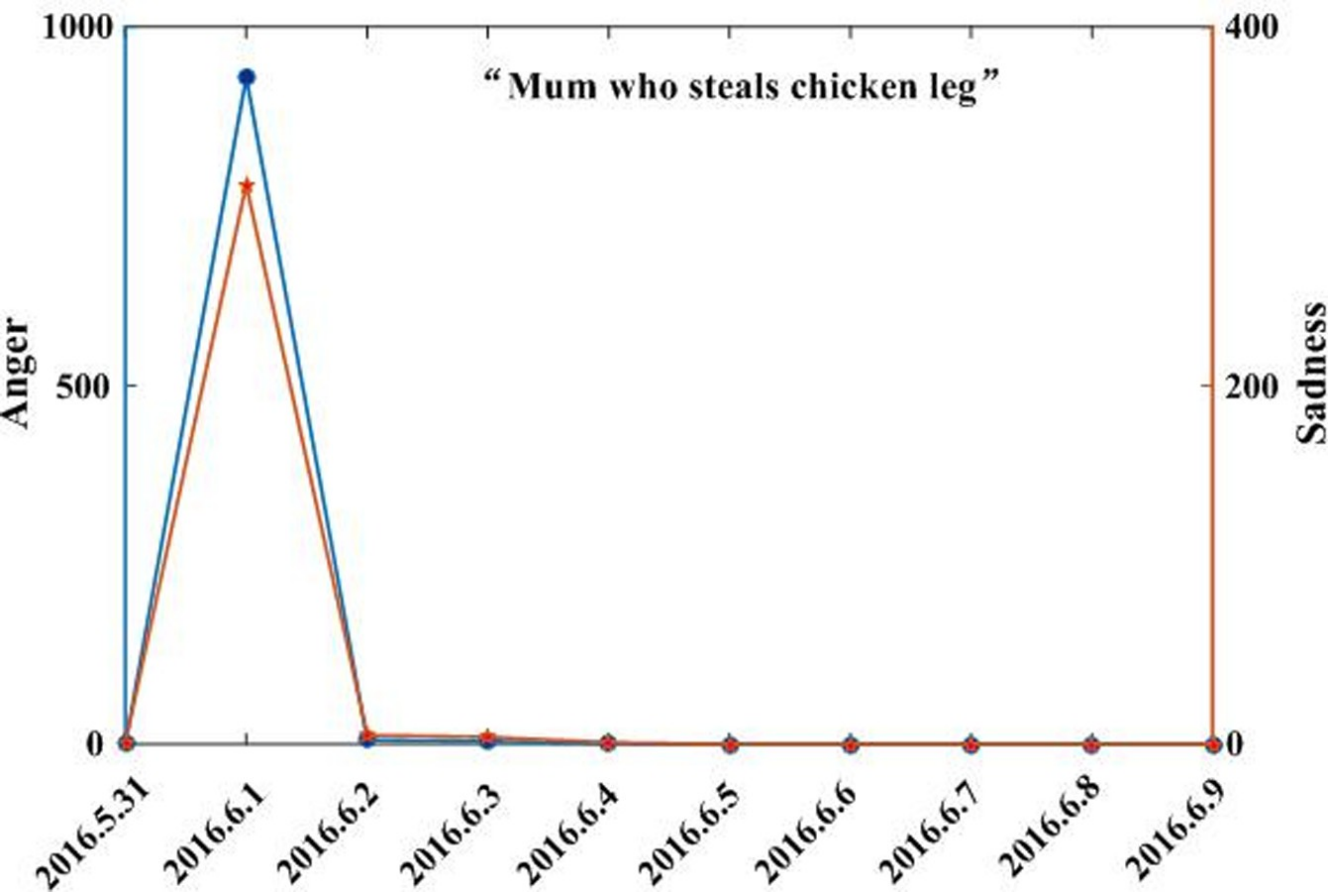
Evolution of emotional trend ratio of the mainstream negative emotions to the “Mum who steals chicken leg” event.

**Fig 9:**
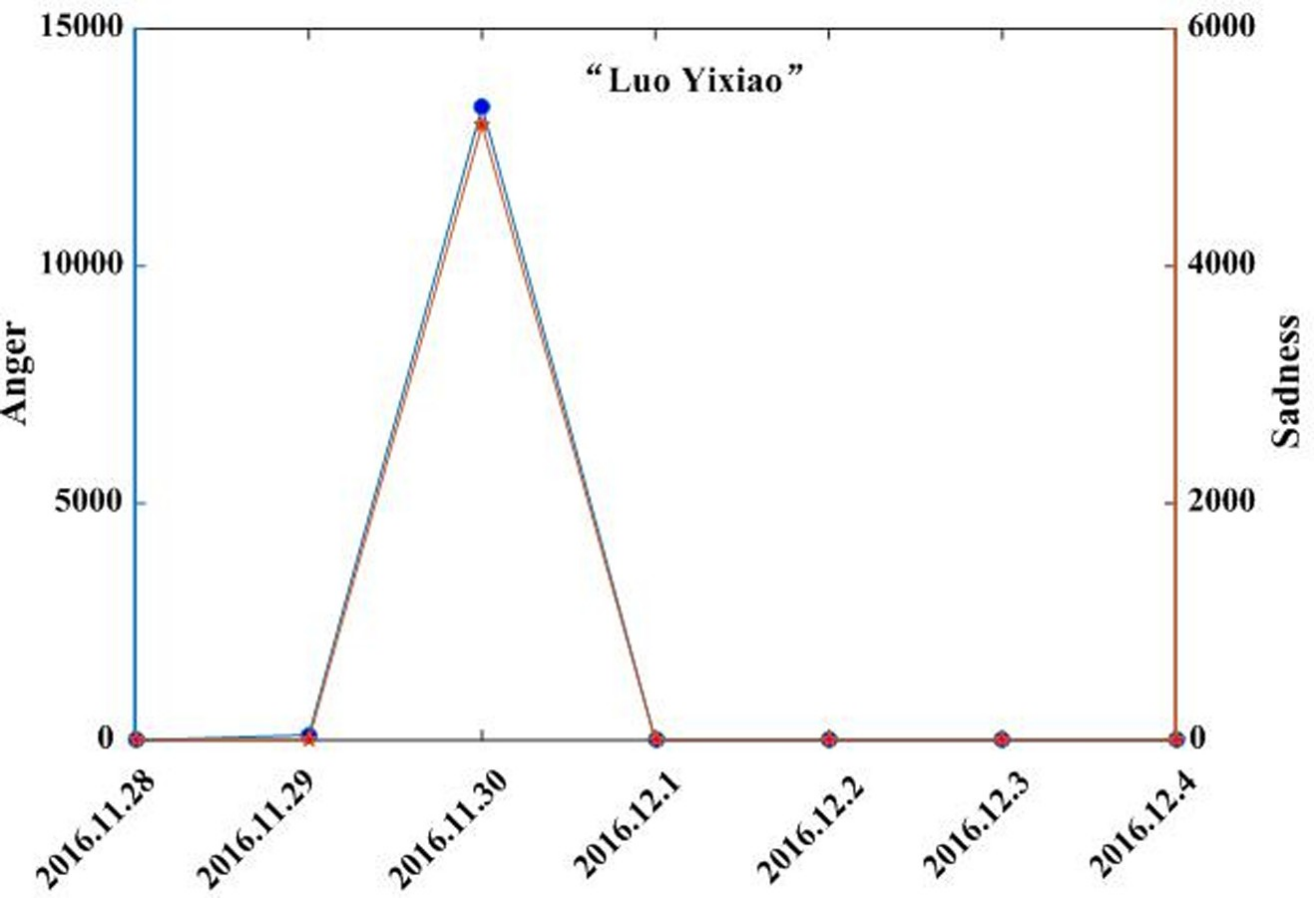
Evolution of emotional trend ratio of the mainstream negative emotions to the “Luo Yixiao” event.

Based on the emotional coverage, *EC*_*ti*_, of the two mainstream emotions of “anger” and “sadness,” we further calculated the emotional trend ratio, *ET*_*t*_, for these two emotions. Figs 6, 7, 8 and 9 show the changes in the *ET*_*t*_ values of the two mainstream emotions during the evolution of the four events. We used two Y-axes to clearly demonstrate the changes in the *ET*_*t*_ values of the two mainstream emotions in the process of event evolution. The left Y-axis represents the *ET*_*t*_ value of “anger” and the right Y-axis represents that of “sadness”. The experimental results show that in the process of event evolution, the *ET*_*t*_ value of the negative mainstream emotion has a significant pulse mutation in a specific time window. These specific time windows occur within 1–3 days before the event. This shows that before the outbreak of the event, the negative mainstream emotions of netizens undergo a significant mutation, specifically manifested such that more netizens from various regions resonated with this negative emotion and expressed their views on the event by publishing more posts. This mutation leads to the breakout of the event in a short period of time. The results show that identifying the mainstream negative emotions of netizens and monitoring the sudden changes in the emotional intensity can help in predicting the outbreak of events.

By observing the evolutionary trend of emotional coverage under the four events, we found that when the value of the emotional trend ratio, *ET*_*t*_, of mainstream emotions exceeded 150, all four events erupted and evolved into more controversial events in the subsequent time windows. Therefore, we set the value of the threshold δ to 150 in this study. When the value of the emotional trend ratio *ET*_*t*_ of the mainstream emotions exceeds the threshold, δ, the event will erupt in the following time windows. The time window when *ET*_*t*_ first exceeds the threshold δ is defined as the critical outbreak time window of the event. Considering the event of “Wei Zexi” as an example, it can be concluded that the critical outbreak time window of the event is May 1, 2016. The incident erupted on May 3, 2016 and reached its peak. For the other three events, i.e., “Nanny arson in Hangzhou,” “Mum who steals chicken leg,” and “Luo Yixiao,” the critical outbreak time windows identified by this method are June 23, 2016, June 1, 2016, and November 30, 2016, respectively. These three events broke out on June 26, 2016, June 3, 2016, and December 1, 2016, respectively, in the future time window near the identified critical time window.

According to the recognition results of the four representative events, the method proposed in this study could effectively identify the critical outbreak time window of the four events. In comparison to the method that uses the number of event-related microblog texts as an indicator of event outbreak, the proposed method can predict event outbreaks at least one day in advance, thereby accomplishing the purpose of prescreening event outbreaks. This prescreen can give more time to the authorities to adopt reasonable prevention, control, and supervision strategies before the event really breaks out with the aim of reducing the risks and hazards caused by the event.

## Conclusions and future work

### Conclusions

The effective identification of the critical time window before the outbreak of an event is of great significance for the relevant government departments to prevent and control the events in advance and reduce the losses caused by the events. During the evolution of the public opinion toward an event, the emotional response of netizens to the event plays an important role in forecasting and guiding the development trend of events.

Microblogs are the mainstream social platforms for Chinese netizens to express their views and exchange information, as well as the main platform for spreading event-related network public opinions in China. Taking four typical events that occurred in China in the recent years as an example, this study explored the possibility of identifying the critical outbreak time window before the occurrence of the events. Through the discussion of the subtle emotional tendencies of netizens toward the events, this study revealed that the different emotion categories of netizens have different impacts on the evolution of events. Some emotions, especially some negative emotions, play a direct guiding role in the development of an event, and can also predict the development trend of the public opinion toward the event. These emotions are the mainstream emotions reflecting the attitudes and views of most netizens. In this study, we propose several definitions to determine the mainstream emotions of an event by considering the text coverage and region coverage characteristics of each emotional category. By detecting the evolution of mainstream emotions, we found that the sudden increase in the emotional coverage characteristics of mainstream emotions can appropriately predict the outbreak of events in the future time window. The surge in the impact intensity of mainstream negative emotion shows that emotions induce more emotional resonance among netizens, which makes the event more controversial and has a more negative impact on the social life. Through the empirical analysis of the four events, it was found that the critical outbreak time window determined by the proposed method lies just before the real outbreak of the event, which shows the effectiveness of the proposed method. Simultaneously, it proves that emotional analysis is a valuable method for analyzing the development trend of events. The research work and conclusions of this study can help in issuing early warnings related to controversial events and help relevant management departments in implementing timely measures to monitor and intervene in the development of events. However, there are still some limitations to this work.

First, at present, the current research work can only help relevant management departments in obtaining information about the time of intervention; it cannot help in determining the appropriate object of intervention. The dissemination and development of events involve the participation of numerous netizens, but the roles and importance of different netizens are different. To help the government implement more targeted and effective intervention measures, it is necessary to identify the key disseminators affecting the dissemination of public opinion and focus on their supervision and intervention. Second, owing to the limitation of data access, we only selected four Chinese cases to complete the study. Due to regional/cultural differences, the conclusions of this study may not be directly extrapolated to case studies in other countries or regions. Third, this study selected 150 as the warning threshold, which is a conservative choice. If the emotional intensity of a certain time window reaches 150 times the previous average, it is considered a strong emotional mutation. For other cases, whether the threshold value determined in this study is appropriate is a problem worthy of discussion.

In addition, this study only selected four Chinese cases for analysis. Because microblogs are the most mainstream social media platform in China, all data included in this study were obtained from the Sina Weibo platform. Owing to the limitations of experimental conditions, this study does not discuss whether the particularity of social media platforms (such as Twitter) will affect the experimental results. However, many previous studies based on Twitter pointed out that the response of netizens is an important factor affecting the development of events. The research performed in this study is a further in-depth analysis of these conclusions. Our experimental results showed that the mutation of the negative mainstream emotion intensity of netizens indicates an outbreak of events. This conclusion further elucidates that the changes in the emotions of netizens play an important role in the development of events, and can be used as an important guiding factor for issuing early warning for events. This conclusion provides valuable information for researchers to explore the impact of emotions on the development of events.

### Future work

In future work, we plan to further explore the applicability of the proposed method in more domestic and foreign cases. Through the analysis of these more extensive cases, we can explore whether regional/cultural differences will affect the early warning research of controversial events based on emotional analysis. Meanwhile, more case studies can also help in determining a more universal warning threshold, which can help in further promoting the application of the method in practice.

In addition to effectively determining the time factor of event warning, identifying the core communicators in the evolution process of events can also help to better monitor the development trend of events. Combined with the communication and reply relationship between microblog texts, a communication network of public opinion on events can be constructed. Through the analysis of the network structure, the key communicators in the process of public opinion dissemination can be identified. Combined with the multi-emotional analysis technology used in this study, we can identify the emotional tendencies of key communicators. In the process of event evolution, the emotional holders who are consistent with the mainstream negative emotions are considered as important personnel to be monitored. The combination of the identification of the outbreak time of an event and the identification of key communicators can help the relevant management departments to intervene in the events in a timely manner, restrain the further deterioration of events, and reduce the harmful impacts of events on social life.

## Supporting information

S1 File (DOCX)

S1 Data (XLS)
