# Supplementary material for: Identifying critical outbreak time window of controversial events based on sentiment analysis
Source: PLoS One. 2020 Oct 29;15(10):e0241355. doi: 10.1371/journal.pone.0241355 (PMC7595406; doi:10.1371/journal.pone.0241355)
Supplement: S1 File — (DOCX) [file pone.0241355.s001.docx]

**S1. “Wei Zexi” incident**

The “Wei Zexi” incident occurred in 2016. Wei Zexi, suffering from malignant soft tissue tumor, searched Baidu for biological immunotherapy at the second Beijing Hospital of the Chinese People’s Armed Police Force; he received biological immune therapy at the hospital. However, Wei eventually died because his condition deteriorated. After the event, Baidu promotion and “Putian hospital” were exposed and became the focus of attention for the whole Chinese population, which triggered extensive discussion on China’s medical and medical supervision strategies.

**Important evolution nodes in the “Wei Zexi” incident**

April 12, 2016: Wei Zexi died at his home in Xianyang, Shanxi, at the age of 21.

April 28, 2016: Baidu promotion and “Putian Department” hospitals were exposed and become the focus of attention in China, and triggered extensive discussion on China’s medical problems and medical supervision. Baidu, in response to the incident, said that the second Beijing Hospital of the Chinese People’s Armed Police Force is a public tertiary hospital with complete qualifications.

May 1, 2016: Baidu once again responded that it was actively submitting an application for examination to the relevant authorities of the license issuing unit and the armed police headquarters, hoping that the relevant authorities would notice it and launch an investigation immediately.

May 2, 2016: Baidu responded for the third time that it welcomed the establishment of a joint investigation team by the state cyber information office and would fully cooperate with the competent authorities in the investigation and accept supervision.

May 3, 2016: The state health and Family Planning Commission, Health Bureau of the Logistics Support Department of the Central Military Commission, and Health Bureau of the Logistics Department of the Armed Police Force jointly conducted an investigation into the second Beijing Hospital of the Chinese People’s Armed Police Force.

May 4, 2016: The second Beijing Hospital of the Chinese People’s Armed Police Force announced that it would stop diagnosis for rectification.

**S2. “Nanny arson in Hangzhou” incident**

The “Nanny arson in Hangzhou” incident occurred in 2017. On June 22, 2017, a fire broke out in the blue Qianjiang community in the east of Hangzhou city. In this fire, a mother and her three children died. After investigation by the public security organization, it was found to be a case of arson. The nanny, named Mo, of the three victims was suspected of major crimes. After examination, Mo confessed that at approximately 5:00 a.m., she used a lighter to light the articles in the living room, which led to the tragedy. For some time, the incident spread on the internet, which triggered heated discussions among netizens about the good and evil of human nature, domestic service quality and supervision, fire control channels, property fire control, and other issues.

**Important evolution nodes in the “Nanny arson in Hangzhou” incident**

June 22, 2017: A fire broke out in a house in the blue Qianjiang community in the east of Hangzhou City, killing the hostess and her three children. The family’s nanny, Mo, was a major suspect in this incident. News of this incident spread on the internet, which caused netizens to discuss the good and evil of human nature, the quality and supervision of domestic service, and how to escape from fire.

June 28, 2017: Hangzhou Public Security Bureau applied to Hangzhou People’s Procuratorate for approval to arrest Mo for arson and theft.

June 29, 2017: Netizens discussed the property fire protection issues of the green city property service group. The green city property service group responded that after the incident, a phenomenon was observed wherein the security personnel altered the inspection records of fire extinguishers, and the company’s management would conduct comprehensive reflection and self-rectification.

July 1, 2017: Hangzhou People’s Procuratorate of Zhejiang Province approved the arrest of suspect Mo for arson and theft.

**S3. “Mum who steals chicken leg”** **incident**

The “Mum who steals chicken leg” incident occurred in 2016. On the eve of children’s day in 2016, a mother stole some coarse grain, a chicken leg, and two children’s books from the supermarket. After being arrested, she told the police that they were for her sick child. Both twins were diagnosed with kidney disease. To increase nutrition for the child, but also want to give the child a children’s day gift, the mother in helpless circumstances to implement theft. This was “the most heartbreaking gift for children’s day” and it gave rise to heated discussion among netizens on the social assistance mechanism.

**Important evolution nodes in the “Mum who steals chicken leg” incident**

May 31, 2016: A mother stole some coarse grain, a chicken leg, and two children’s books from the supermarket. After being arrested, she told the police that she committed theft for her sick child.

Early in the morning of June 1, 2016: The police forwarded the incident to a WeChat circle of friends, causing widespread concern among netizens. A “micro public welfare” donation channel was started for the mother in less than three hours. It received more than 300,000 RMB in donations from netizens, making it the fastest public welfare relief project for children’s day in 2016.

June 2, 2016: Netizens set off a “moral debate” on this matter, which resulted in the mother apologizing under moral pressure.

June 3, 2016: Netizens began to reflect on improving the social assistance system. Netizens generally believe that the prevention of the next “Mum who steals chicken leg” is the real problem to be considered.

**S4. “Luo Yixiao” incident**

The “Luo Yixiao” incident occurred in 2016. At the end of November 2016, a local writer in Shenzhen, China, published an article titled “Luo Yixiao, you stop for me” in his circle of friends. The article claimed that Luo Yixiao, the author’s five-year-old daughter, was seriously ill and required a significant amount of money for paying the medical expenses. Instead of accepting public welfare donations, the father chose to sell articles. If this article is forwarded once, he will receive a donation of 1 yuan for the treatment of his daughter, Luo Yixiao. However, some netizens pointed out that the incident was a marketing hype, which caused heated discussion on the event. The attitude of netizens toward the incident also changed from the initial burst of collective love to the accusation of “bloody marketing,” followed by the moral bottom line of torturing humanity.

**Important evolution nodes in the “Luo Yixiao” incident**

November 28, 2016: A writer named Rolle published an article “Luo Yixiao, you stop for me,” which garnered widespread attention on the internet. Netizens began to forward the article and solicit donations.

November 29, 2016: Rolle published the article “Don’t let children get lost because of money.” Thus far, Rolle had received donation funds of more than 120,000 RMB from netizens.

November 30, 2016: Netizens questioned Rolle’s property and his daughter’s medical expenses. The event itself was denounced by netizens as “bloody marketing” and Rolle was soon involved in the whirlpool of public opinion.

December 1, 2016: Shenzhen Civil Affairs Bureau intervened in the investigation.

December 3, 2016: Rolle returned all fundraising money to the netizens.
